# *N*-(2,6-Dimethylphenyl)-*N*′-propanoyl­thiourea

**DOI:** 10.1107/S1600536812009233

**Published:** 2012-03-07

**Authors:** Mohd Sukeri Mohd Yusof, Siti Fatimah Abdul Mutalib, Suhana Arshad, Ibrahim Abdul Razak

**Affiliations:** a Department of Chemical Sciences, Faculty of Science and Technology, Universiti Malaysia Terengganu, Mengabang Telipot, 21030 Kuala Terengganu, Malaysia; b School of Physics, Universiti Sains Malaysia, 11800 USM, Penang, Malaysia

## Abstract

In the title compound, C_12_H_16_N_2_OS, an intra­molecular N—H⋯O hydrogen bond forms an *S*(6) ring motif. The propionyl­thio­urea group is approximately planar [with a maximum deviation of 0.135 (2) Å] and forms a dihedral angle of 83.39 (7)° with the benzene ring. In the crystal, mol­ecules are linked by pairs of N—H⋯S hydrogen bonds, forming centrosymmetric dimers and generating *R* ^2^_2_(8) ring motifs.

## Related literature

For related structures, see: Yamin & Othman (2008 ▶); Usman *et al.* (2002 ▶); Sultana *et al.* (2007 ▶). For hydrogen-bond motifs, see: Bernstein *et al.* (1995 ▶). For the stability of the temperature controller used in the data collection, see: Cosier & Glazer (1986 ▶).
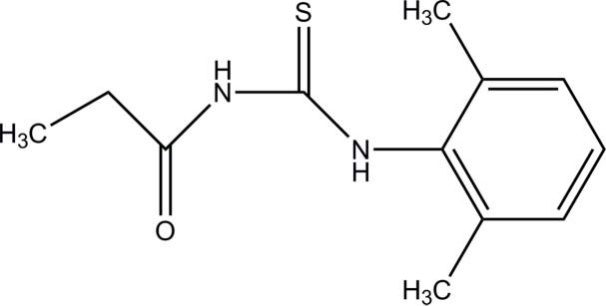


## Experimental

### 

#### Crystal data

C_12_H_16_N_2_OS
*M* *_r_* = 236.33
Triclinic, 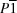

*a* = 7.8069 (3) Å
*b* = 8.4770 (3) Å
*c* = 10.1426 (3) Å
α = 103.782 (2)°
β = 90.342 (2)°
γ = 109.928 (2)°
*V* = 610.07 (4) Å^3^
*Z* = 2
Mo *K*α radiation
μ = 0.25 mm^−1^
*T* = 100 K
0.23 × 0.18 × 0.06 mm

#### Data collection

Bruker SMART APEXII CCD area-detector diffractometer
Absorption correction: multi-scan (*SADABS*; Bruker, 2009) ▶ *T*_min_ = 0.946, *T*_max_ = 0.985
6225 measured reflections
3211 independent reflections
2664 reflections with *I* > 2σ(*I*)
*R*_int_ = 0.025

#### Refinement

*R*[*F* ^2^ > 2σ(*F* ^2^)] = 0.040
*wR*(*F* ^2^) = 0.100
*S* = 1.00
3211 reflections
156 parameters
H atoms treated by a mixture of independent and constrained refinement
Δρ_max_ = 0.40 e Å^−3^
Δρ_min_ = −0.31 e Å^−3^

### 

Data collection: *APEX2* (Bruker, 2009) ▶; cell refinement: *SAINT* (Bruker, 2009) ▶; data reduction: *SAINT* ▶; program(s) used to solve structure: *SHELXTL* (Sheldrick, 2008 ▶); program(s) used to refine structure: *SHELXTL*; molecular graphics: *SHELXTL*; software used to prepare material for publication: *SHELXTL* and *PLATON* (Spek, 2009 ▶).

## Supplementary Material

Crystal structure: contains datablock(s) global, I. DOI: 10.1107/S1600536812009233/lh5423sup1.cif

Structure factors: contains datablock(s) I. DOI: 10.1107/S1600536812009233/lh5423Isup2.hkl

Supplementary material file. DOI: 10.1107/S1600536812009233/lh5423Isup3.cml

Additional supplementary materials:  crystallographic information; 3D view; checkCIF report

## Figures and Tables

**Table 1:** Hydrogen-bond geometry (Å, °)

*D*—H⋯*A*	*D*—H	H⋯*A*	*D*⋯*A*	*D*—H⋯*A*
N2—H1*N*2⋯O1	0.85 (2)	1.98 (2)	2.6661 (19)	138 (2)
N1—H1*N*1⋯S1^i^	0.87 (2)	2.54 (2)	3.3765 (15)	162.0 (16)

Symmetry code: (i) 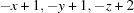.

**Fig. 1.**
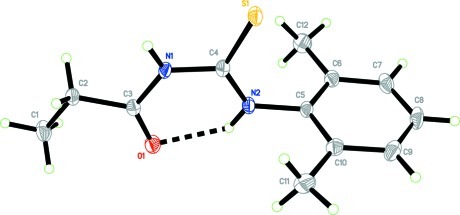
The molecular structure of the title compound with 30% probability displacement ellipsoids.

**Fig. 2.**
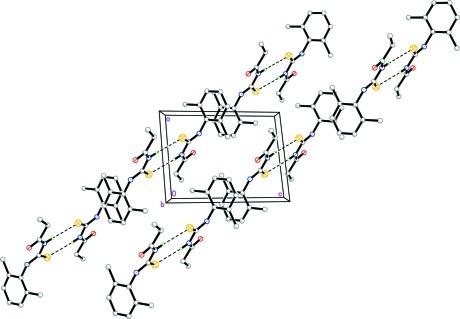
The crystal packing of the title compound. The H atoms not involved in the intermolecular interactions (dashed lines) have been omitted for clarity.

## supplementary crystallographic information

### Comment

The title compound is analogous to N-propionylthiourea, (Yamin & Othman, 2008) except that the hydogen atom at the N terminal atom is replaced by a 2,6-dimethylphenyl group.

In the molecular structure (Fig. 1), an intramolecular N2—H1N2···O1 hydrogen bond (Table 1) generates an S(6) ring motif (Bernstein et al., 1995). The propionylthiourea group (S1/N1/N2/O1/C1-C4) is approximately planar (with a maximum deviation of 0.135 (2)Å for C1) and forms a dihedral angle of 83.39 (7)° with the benzene ring (C5-C10). The bond lengths and angles are within normal ranges and are comparable to related structures (Usman et al., 2002; Sultana et al., 2007).

The crystal packing is shown in Fig. 2. The molecules are linked by pairs of intermolecular N1—H1N1···S1^i^ hydrogen bonds (Table 1) to form dimers, generating R^2^_2_(8) ring motifs (Bernstein et al., 1995).

### Experimental

To a stirring acetone solution (75 ml) of propionyl chloride (2.42 g, 0.03 mol) and ammonium thiocyanate (2.0 g, 0.03 mol), 2,6-dimethylaniline (3.64 g, 0.03 mol) in 40 ml of acetone was added dropwise. The mixture was refluxed reflux for 1 h. The resulting solution was poured into a beaker containing ice blocks. The white precipitate was filtered off and washed with distilled water and cold ethanol before being dried under vacuum. Good quality crystals were obtained by recrystallization from DMSO.

### Refinement

N-bound H atoms were located from the difference map and refined freely, [N–H = 0.85 (2) and 0.87 (2) Å]. The remaining H atoms were positioned geometrically [C–H = 0.95-0.99 Å] and refined using a riding model with *U*_iso_(H) = 1.2 or 1.5 *U*_eq_(C). A rotating group model was applied to the methyl groups.

### Crystal data

**Table:**

C_12_H_16_N_2_OS	*Z* = 2
*M**_r_* = 236.33	*F*(000) = 252
Triclinic, *P*1	*D*_x_ = 1.287 Mg m^−^^3^
Hall symbol: -P 1	Mo *K*α radiation, λ = 0.71073 Å
*a* = 7.8069 (3) Å	Cell parameters from 2626 reflections
*b* = 8.4770 (3) Å	θ = 2.8–30.1°
*c* = 10.1426 (3) Å	µ = 0.25 mm^−^^1^
α = 103.782 (2)°	*T* = 100 K
β = 90.342 (2)°	Plate, colourless
γ = 109.928 (2)°	0.23 × 0.18 × 0.06 mm
*V* = 610.07 (4) Å^3^	

### Data collection

**Table:**

Bruker SMART APEXII CCD area-detector diffractometer	3211 independent reflections
Radiation source: fine-focus sealed tube	2664 reflections with *I* > 2σ(*I*)
Graphite monochromator	*R*_int_ = 0.025
φ and ω scans	θ_max_ = 29.0°, θ_min_ = 2.1°
Absorption correction: multi-scan (*SADABS*; Bruker, 2009)	*h* = −10→5
*T*_min_ = 0.946, *T*_max_ = 0.985	*k* = −11→11
6225 measured reflections	*l* = −13→13

### Refinement

**Table:**

Refinement on *F*^2^	Primary atom site location: structure-invariant direct methods
Least-squares matrix: full	Secondary atom site location: difference Fourier map
*R*[*F*^2^ > 2σ(*F*^2^)] = 0.040	Hydrogen site location: inferred from neighbouring sites
*wR*(*F*^2^) = 0.100	H atoms treated by a mixture of independent and constrained refinement
*S* = 1.00	*w* = 1/[σ^2^(*F*_o_^2^) + (0.0263*P*)^2^ + 0.6043*P*] where *P* = (*F*_o_^2^ + 2*F*_c_^2^)/3
3211 reflections	(Δ/σ)_max_ = 0.001
156 parameters	Δρ_max_ = 0.40 e Å^−^^3^
0 restraints	Δρ_min_ = −0.31 e Å^−^^3^

### Special details

**Table:**

Experimental. The crystal was placed in the cold stream of an Oxford Cryosystems Cobra open-flow nitrogen cryostat (Cosier & Glazer, 1986) operating at 100.0 (1) K.
Geometry. All e.s.d.'s (except the e.s.d. in the dihedral angle between two l.s. planes) are estimated using the full covariance matrix. The cell e.s.d.'s are taken into account individually in the estimation of e.s.d.'s in distances, angles and torsion angles; correlations between e.s.d.'s in cell parameters are only used when they are defined by crystal symmetry. An approximate (isotropic) treatment of cell e.s.d.'s is used for estimating e.s.d.'s involving l.s. planes.
Refinement. Refinement of *F*^2^ against ALL reflections. The weighted *R*-factor *wR* and goodness of fit *S* are based on *F*^2^, conventional *R*-factors *R* are based on *F*, with *F* set to zero for negative *F*^2^. The threshold expression of *F*^2^ > σ(*F*^2^) is used only for calculating *R*-factors(gt) *etc*. and is not relevant to the choice of reflections for refinement. *R*-factors based on *F*^2^ are statistically about twice as large as those based on *F*, and *R*- factors based on ALL data will be even larger.

### Fractional atomic coordinates and isotropic or equivalent isotropic displacement parameters (Å^2^)

**Table:**

	*x*	*y*	*z*	*U*_iso_*/*U*_eq_	
S1	0.29160 (6)	0.31937 (5)	0.83787 (4)	0.02011 (12)	
N1	0.48198 (18)	0.65151 (17)	0.86755 (14)	0.0150 (3)	
H1N1	0.537 (3)	0.634 (2)	0.934 (2)	0.018 (5)*	
N2	0.23611 (18)	0.53214 (18)	0.70040 (14)	0.0159 (3)	
H1N2	0.269 (3)	0.634 (3)	0.690 (2)	0.032 (6)*	
O1	0.45702 (17)	0.86360 (15)	0.77368 (12)	0.0218 (3)	
C1	0.7853 (2)	1.1145 (2)	0.90542 (19)	0.0243 (4)	
H1A	0.8956	1.1898	0.9662	0.036*	
H1B	0.6927	1.1697	0.9161	0.036*	
H1C	0.8158	1.0959	0.8107	0.036*	
C2	0.7107 (2)	0.9413 (2)	0.94142 (17)	0.0184 (3)	
H2A	0.8052	0.8867	0.9313	0.022*	
H2B	0.6838	0.9612	1.0380	0.022*	
C3	0.5388 (2)	0.8192 (2)	0.85224 (16)	0.0153 (3)	
C4	0.3335 (2)	0.5086 (2)	0.79737 (16)	0.0152 (3)	
C5	0.0741 (2)	0.3969 (2)	0.62443 (16)	0.0153 (3)	
C6	0.0916 (2)	0.2808 (2)	0.50703 (17)	0.0187 (3)	
C7	−0.0683 (3)	0.1516 (2)	0.43662 (18)	0.0229 (4)	
H7A	−0.0605	0.0696	0.3569	0.027*	
C8	−0.2383 (2)	0.1413 (2)	0.48146 (19)	0.0251 (4)	
H8A	−0.3457	0.0529	0.4322	0.030*	
C9	−0.2519 (2)	0.2590 (2)	0.59732 (19)	0.0239 (4)	
H9A	−0.3691	0.2508	0.6270	0.029*	
C10	−0.0956 (2)	0.3906 (2)	0.67187 (17)	0.0188 (3)	
C11	−0.1090 (3)	0.5156 (2)	0.79955 (19)	0.0254 (4)	
H11A	−0.0389	0.5064	0.8760	0.038*	
H11B	−0.2376	0.4883	0.8180	0.038*	
H11C	−0.0594	0.6340	0.7884	0.038*	
C12	0.2756 (3)	0.2913 (2)	0.45815 (19)	0.0252 (4)	
H12A	0.3586	0.2987	0.5341	0.038*	
H12B	0.3259	0.3946	0.4234	0.038*	
H12C	0.2622	0.1874	0.3852	0.038*	

### Atomic displacement parameters (Å^2^)

**Table:**

	*U*^11^	*U*^22^	*U*^33^	*U*^12^	*U*^13^	*U*^23^
S1	0.0208 (2)	0.01442 (19)	0.0210 (2)	0.00006 (15)	−0.00808 (16)	0.00615 (15)
N1	0.0141 (6)	0.0134 (6)	0.0152 (6)	0.0021 (5)	−0.0051 (5)	0.0037 (5)
N2	0.0152 (7)	0.0128 (6)	0.0165 (7)	0.0013 (5)	−0.0038 (5)	0.0033 (5)
O1	0.0238 (6)	0.0167 (6)	0.0229 (6)	0.0041 (5)	−0.0069 (5)	0.0061 (5)
C1	0.0232 (9)	0.0169 (8)	0.0260 (9)	−0.0010 (7)	−0.0023 (7)	0.0052 (7)
C2	0.0179 (8)	0.0140 (7)	0.0199 (8)	0.0028 (6)	−0.0038 (6)	0.0025 (6)
C3	0.0150 (7)	0.0145 (7)	0.0141 (7)	0.0037 (6)	0.0009 (6)	0.0019 (5)
C4	0.0135 (7)	0.0154 (7)	0.0142 (7)	0.0032 (6)	0.0002 (6)	0.0019 (6)
C5	0.0152 (7)	0.0131 (7)	0.0153 (7)	0.0019 (6)	−0.0044 (6)	0.0043 (6)
C6	0.0197 (8)	0.0171 (8)	0.0179 (8)	0.0044 (6)	−0.0033 (6)	0.0051 (6)
C7	0.0283 (9)	0.0165 (8)	0.0179 (8)	0.0020 (7)	−0.0081 (7)	0.0025 (6)
C8	0.0212 (9)	0.0211 (9)	0.0260 (9)	−0.0028 (7)	−0.0126 (7)	0.0091 (7)
C9	0.0161 (8)	0.0267 (9)	0.0289 (9)	0.0033 (7)	−0.0035 (7)	0.0134 (7)
C10	0.0192 (8)	0.0195 (8)	0.0191 (8)	0.0070 (6)	−0.0011 (6)	0.0072 (6)
C11	0.0220 (9)	0.0285 (9)	0.0275 (9)	0.0114 (7)	0.0038 (7)	0.0069 (7)
C12	0.0263 (9)	0.0268 (9)	0.0207 (9)	0.0096 (7)	0.0026 (7)	0.0024 (7)

### Geometric parameters (Å, º)

**Table:**

S1—C4	1.6756 (16)	C5—C10	1.400 (2)
N1—C3	1.385 (2)	C6—C7	1.397 (2)
N1—C4	1.393 (2)	C6—C12	1.503 (2)
N1—H1N1	0.87 (2)	C7—C8	1.386 (3)
N2—C4	1.331 (2)	C7—H7A	0.9500
N2—C5	1.445 (2)	C8—C9	1.380 (3)
N2—H1N2	0.85 (2)	C8—H8A	0.9500
O1—C3	1.219 (2)	C9—C10	1.401 (2)
C1—C2	1.517 (2)	C9—H9A	0.9500
C1—H1A	0.9800	C10—C11	1.495 (2)
C1—H1B	0.9800	C11—H11A	0.9800
C1—H1C	0.9800	C11—H11B	0.9800
C2—C3	1.511 (2)	C11—H11C	0.9800
C2—H2A	0.9900	C12—H12A	0.9800
C2—H2B	0.9900	C12—H12B	0.9800
C5—C6	1.393 (2)	C12—H12C	0.9800
			
C3—N1—C4	127.85 (14)	C5—C6—C7	117.67 (16)
C3—N1—H1N1	117.2 (13)	C5—C6—C12	121.57 (15)
C4—N1—H1N1	114.7 (13)	C7—C6—C12	120.75 (16)
C4—N2—C5	122.62 (13)	C8—C7—C6	120.93 (17)
C4—N2—H1N2	116.3 (15)	C8—C7—H7A	119.5
C5—N2—H1N2	120.9 (15)	C6—C7—H7A	119.5
C2—C1—H1A	109.5	C9—C8—C7	120.23 (16)
C2—C1—H1B	109.5	C9—C8—H8A	119.9
H1A—C1—H1B	109.5	C7—C8—H8A	119.9
C2—C1—H1C	109.5	C8—C9—C10	121.04 (17)
H1A—C1—H1C	109.5	C8—C9—H9A	119.5
H1B—C1—H1C	109.5	C10—C9—H9A	119.5
C3—C2—C1	112.25 (14)	C5—C10—C9	117.38 (16)
C3—C2—H2A	109.2	C5—C10—C11	121.28 (15)
C1—C2—H2A	109.2	C9—C10—C11	121.31 (16)
C3—C2—H2B	109.2	C10—C11—H11A	109.5
C1—C2—H2B	109.2	C10—C11—H11B	109.5
H2A—C2—H2B	107.9	H11A—C11—H11B	109.5
O1—C3—N1	122.77 (15)	C10—C11—H11C	109.5
O1—C3—C2	123.23 (14)	H11A—C11—H11C	109.5
N1—C3—C2	114.00 (14)	H11B—C11—H11C	109.5
N2—C4—N1	117.11 (14)	C6—C12—H12A	109.5
N2—C4—S1	124.53 (12)	C6—C12—H12B	109.5
N1—C4—S1	118.36 (12)	H12A—C12—H12B	109.5
C6—C5—C10	122.74 (15)	C6—C12—H12C	109.5
C6—C5—N2	119.40 (14)	H12A—C12—H12C	109.5
C10—C5—N2	117.85 (15)	H12B—C12—H12C	109.5
			
C4—N1—C3—O1	2.3 (3)	C10—C5—C6—C12	179.71 (15)
C4—N1—C3—C2	−177.72 (15)	N2—C5—C6—C12	0.8 (2)
C1—C2—C3—O1	−9.3 (2)	C5—C6—C7—C8	0.9 (2)
C1—C2—C3—N1	170.72 (14)	C12—C6—C7—C8	179.91 (16)
C5—N2—C4—N1	−177.10 (14)	C6—C7—C8—C9	−0.2 (3)
C5—N2—C4—S1	4.1 (2)	C7—C8—C9—C10	−0.1 (3)
C3—N1—C4—N2	2.4 (2)	C6—C5—C10—C9	1.0 (2)
C3—N1—C4—S1	−178.77 (13)	N2—C5—C10—C9	179.92 (14)
C4—N2—C5—C6	−87.4 (2)	C6—C5—C10—C11	179.02 (15)
C4—N2—C5—C10	93.64 (19)	N2—C5—C10—C11	−2.0 (2)
C10—C5—C6—C7	−1.2 (2)	C8—C9—C10—C5	−0.3 (2)
N2—C5—C6—C7	179.82 (14)	C8—C9—C10—C11	−178.34 (16)

### Hydrogen-bond geometry (Å, º)

**Table:**

*D*—H···*A*	*D*—H	H···*A*	*D*···*A*	*D*—H···*A*
N2—H1*N*2···O1	0.85 (2)	1.98 (2)	2.6661 (19)	138 (2)
N1—H1*N*1···S1^i^	0.87 (2)	2.54 (2)	3.3765 (15)	162.0 (16)

Symmetry code: (i) −*x*+1, −*y*+1, −*z*+2.
